# Factors Affecting the Successful Implementation of a Digital Intervention for Health Financing in a Low-Resource Setting at Scale: Semistructured Interview Study With Health Care Workers and Management Staff

**DOI:** 10.2196/38818

**Published:** 2023-01-06

**Authors:** Leon Schuetze, Siddharth Srivastava, Abdallah Mtiba Missenye, Elizeus Josephat Rwezaula, Manfred Stoermer, Manuela De Allegri

**Affiliations:** 1 Heidelberg Institute of Global Health Medical Faculty and University Hospital University of Heidelberg Heidelberg Germany; 2 Swiss Tropical and Public Health Institute (Swiss TPH) Basel Switzerland; 3 University of Basel Basel Switzerland; 4 Kongwa District Council Dodoma United Republic of Tanzania; 5 Health Promotion and System Strengthening Project (HPSS) Dodoma United Republic of Tanzania

**Keywords:** health financing, qualitative, digital health intervention, low-resource setting, strategic purchasing, scale, mobile phone

## Abstract

**Background:**

Digital interventions for health financing, if implemented at scale, have the potential to improve health system performance by reducing transaction costs and improving data-driven decision-making. However, many interventions never reach sustainability, and evidence on success factors for scale is scarce. The Insurance Management Information System (IMIS) is a digital intervention for health financing, designed to manage an insurance scheme and already implemented on a national scale in Tanzania. A previous study found that the IMIS claim function was poorly adopted by health care workers (HCWs), questioning its potential to enable strategic purchasing and succeed at scale.

**Objective:**

This study aimed to understand why the adoption of the IMIS claim function by HCWs remained low in Tanzania and to assess implications for use at scale.

**Methods:**

We conducted 21 semistructured interviews with HCWs and management staff in 4 districts where IMIS was first implemented. We sampled respondents by using a maximum variation strategy. We used the framework method for data analysis, applying a combination of inductive and deductive coding to organize codes in a socioecological model. Finally, we related emerging themes to a framework for digital health interventions for scale.

**Results:**

Respondents appreciated IMIS’s intrinsic software characteristics and technical factors and acknowledged IMIS as a valuable tool to simplify claim management. Human factors, extrinsic ecosystem, and health care ecosystem were considered as barriers to widespread adoption.

**Conclusions:**

Digital interventions for health financing, such as IMIS, may have the potential for scale if careful consideration is given to the environment in which they are placed. Without a sustainable health financing environment, sufficient infrastructure, and human capacity, they cannot unfold their full potential to improve health financing functions and ultimately contribute to universal health coverage.

## Introduction

Universal health coverage (UHC) means that every individual has access to needed health care without risking financial hardship. The World Health Organization considers digital technologies as an essential enabling factor to advance the UHC agenda and defines digital health very broadly as “the field of knowledge and practice associated with the development and use of digital technologies to improve health” [1]. The rapid increase in mobile phone availability and network coverage in low- and middle-income countries (LMICs) has enabled a cornucopia of digital health interventions (DHIs) to emerge over the last decade. These interventions are expected to improve health outcomes by increasing efficiency or improving the quality of care [2-4]. The enthusiasm toward digitization in the health sector extends to health financing, widely recognized as a key area of health system reform toward UHC. In particular, mobile money services, which enable resource collection at the individual level, have been launched in many countries, especially in sub-Saharan Africa [5,6]. Beyond mobile money, DHIs targeting the other 2 health financing functions, pooling and purchasing, have been implemented. The mobile microhealth insurance scheme, Jamii [7], or the claim management system operated by openRBF [8] provide an example of such developments [9,10]. DHIs applied to health financing have the potential to improve health system performance by reducing transaction costs, enabling strategic purchasing, and ultimately improving data-driven decision-making [9,11].

However, reflecting a widespread lack of evidence on DHIs in general [12,13], evidence on DHIs targeting health financing is particularly scarce, especially in LMICs. Many DHIs are not evaluated at all, with a lot of the existing knowledge stemming from project reports rather than peer-reviewed literature and revolving almost exclusively around mobile money services [9-11]. This lack of knowledge is concerning because relying on potentially flawed data from the inadequate implementation of a digital intervention could result in unintended consequences, driving change away from intended health system objectives, and potentially enhance, rather than reduce, the existing equity gaps [10]. In addition to—and maybe as a result of—the lack of evidence, many DHIs, including those addressing health financing, never reach scale, remain stuck at the pilot phase, or are abandoned after a few years [2,12,14-16]. In response to this, a number of efforts have recently been undertaken to identify ways to promote the scale-up of DHIs [2,15,17]. Scaling up a DHI means to take deliberate efforts for it “to benefit more people and to foster policy and program development on a lasting basis” [2]. Beyond this, successful scale-up requires seamless integration within the health system, sustainable funding, government support, and robust management [15,18]. The Principles for Digital Development [19] list *design for scale* as 1 of the 9 guiding principles for integrating technology into development programs and initiatives such as the Health Data Collaborative [20], Digital Impact Alliance [21], and Digital Square [22] have been launched to support scale-up by enhancing strategic investing, coordination and operational guidance [23]. This push toward scale-up of DHIs has coincided with many LMICs’ efforts to move from small, fragmented health financing systems to sustainable national schemes [24,25]. Nationally scaled-up digital interventions for health financing could potentially play a major role in this transition, for example, by facilitating the management of large-scale insurance schemes, enabling both more efficient collection and pooling, and facilitating a shift from passive to strategic purchasing. The Insurance Management Information System (known as IMIS or CHF [Community Health Fund]–IMIS in Tanzania and openIMIS under a global initiative) is an example of such a health financing digital intervention, born as a small-scale solution for a single setting, but having been expanded to the point of acquiring capacity for scale. Despite its wide application in the field, literature on IMIS is so scarce that a quantitative study performed by the authors of this paper is the only one currently available. The study investigated IMIS’s adoption by primary-level health facilities in its initial implementation setting in Tanzania. Adoption, defined as “the intention, initial decision, or action to try or employ an innovation” [26], is one of the most basic implementation outcomes. The authors estimated IMIS adoption levels for 365 primary-level health facilities in 2017 by comparing the number of expected claims (estimated by combining multiple data sources on use and insurance rates) with the number of IMIS claims for each facility. They revealed a median discrepancy of 77.8% (IQR 32.7-100) between the number of claims expected to be submitted by facilities and the number of claims actually submitted to IMIS, thus suggesting significant and widespread underreporting. This discrepancy could not be sufficiently explained by the factors included in the regression analysis, such as district affiliation, remoteness, number of clients, or insurance enrollment in the catchment area [27]. The findings prompted serious doubts regarding the ability of IMIS to facilitate strategic purchasing under these circumstances, providing impetus for the qualitative study described in this paper.

Although IMIS in Tanzania has surpassed the pilot stage and it has been expanded nationally with government support, its adoption levels are low, and this has raised the question as to whether specific adoption barriers stand in the way of a successful scale-up. The basic underlying assumption is that successful scale-up inevitably relies on the intended users ultimately adopting IMIS. Hence, with this study, we set out to explore the reasons for the suboptimal adoption of IMIS by primary-level health facility staff in Tanzania, with the objective of identifying adoption barriers that are potentially hindering successful scale-up in Tanzania and similar settings.

## Methods

### Study Setting

#### Tanzania Health System

With a total per capita health expenditure of US $36.8 and a gross domestic product of US $62.4 billion per year, Tanzania spends less on health than its neighbors, both in absolute numbers and as percentage of gross domestic product [28,29]. The health system is highly fragmented and heavily reliant on donor funding [24,30], with out-of-pocket payments accounting for 24% of the total health expenditure [28]. Public and private facilities are organized in a multitiered and decentralized structure. Dispensaries make up the primary level of care, providing maternal and child health care, attending uncomplicated deliveries, and offering basic outpatient care. Staffing guidelines indicate that they should be staffed by a clinical officer (CO) with 2 years of medical training, assisted by nurses and other staff. However, owing to staff shortages, recommended staffing levels are rarely reached, and a dispensary may be led by a nurse or a medical attendant without formal medical training [31,32]. Higher levels of care are provided by health centers, followed by district, regional, and national hospitals. Most clients pay fees at the point of service, with only extremely poor individuals, pregnant women, children aged <5 years, and older people being supposedly exempted from payment. As of 2014, 16% of Tanzanians had health insurance [30]. The insurance landscape mainly consists of the National Health Insurance Fund for the formal sector (6.6% coverage) and the CHF and other voluntary schemes for the informal sector with 7.3% coverage in 2013. Owing to CHF design reforms, statistics have changed significantly since then, and these are the most recently published estimates [30]. The CHF were piloted in 1996 and rolled out countrywide in 2001. In 2012, a redesigned version, the improved CHF (iCHF), were piloted in the Dodoma region, introducing IMIS (described in the following section), shifting enrollment from the facilities to designated enrollment officers, and enabling cross-facility and cross-district coverage portability. The pilot was expanded to 2 more regions in 2015, and in 2018 the iCHF were scaled up nationally, covering all public and some private facilities in the country. Membership in the scheme covers up to 6 household members and includes coverage of all primary outpatient services and selected secondary-level services.

#### IMIS and openIMIS

IMIS is a DHI designed to manage all business procedures of a health insurance scheme, from client enrollment to claim reimbursement. By digitizing and linking patient, provider, and payer data, IMIS seeks to increase the efficiency and transparency of health financing operations. The software was developed by the Swiss Tropical and Public Health Institute and first implemented within the iCHF pilot in 2012. Since then, IMIS has been made an open source (released under the name of openIMIS), has received notable political support, and has been promoted as a tool to enable strategic purchasing and contribute to advancing UHC [33]. Since its launch in 2012, it has been scaled up nationally in Tanzania and implemented within a number of health insurance schemes in sub-Saharan Africa and Asia [33]. The software inspired the foundation of the openIMIS global initiative coordinated by the German Agency for International Cooperation, and in 2021, openIMIS was recognized as a Digital Global Good by the Digital Public Goods Alliance [34,35].

The core aspect of IMIS’s capacity to enable strategic purchasing is the claiming function. After attending to an insured client, health care providers digitally submit a claim to the insurance scheme via IMIS. Then, the claims are checked and validated, and payments to facilities are calculated according to a predefined payment method. In the long run, aggregated claims and beneficiary data are expected to enable the insurance scheme to make strategic purchasing decisions about what health services to purchase from which providers and at what price [36].

### Study Design

This study is a qualitative follow-up of a quantitative investigation of IMIS adoption by health care workers (HCWs) in dispensaries in Tanzania [27]. The surprisingly widespread low adoption called for an in-depth investigation of its root causes. We conducted semistructured interviews with HCWs using IMIS in rural dispensaries and with CHF district managers in 4 districts across 2 of the iCHF pilot regions. District managers are, among others, responsible for monthly claim validation and serve as support for facility staff regarding any CHF-related, including IMIS, issues.

### Sampling and Data Collection

**Figure 1:**
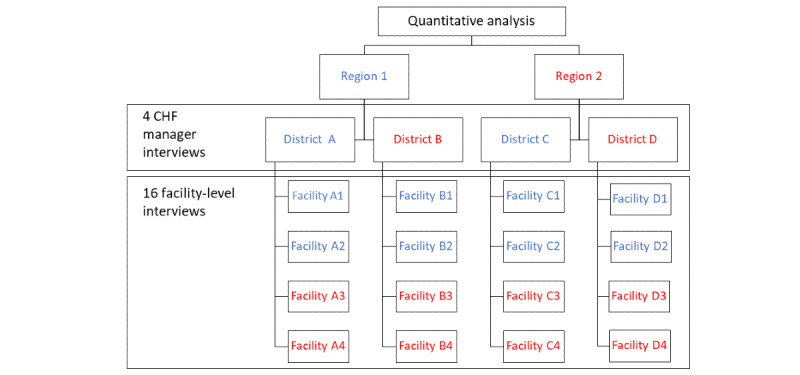
Sampling strategy, wherein blue = high level of adoption in 2017, and red = low level of adoption in 2017. CHF: Community Health Fund.

On the basis of previous quantitative findings that have identified heterogeneity in the adoption of IMIS at the regional and district levels, we applied a maximum variation sampling strategy [37], selecting respondents purposely across regional, district, and facility gradients to reflect this heterogeneity of experiences. Specifically, respondents were selected from the 20% of facilities with the highest and lowest adoption across the rural districts with the highest and lowest overall adoption in the 2 regions (Figure 1). We developed 2 separate interview guides for HCWs and managers (Multimedia Appendices 1 and 2). Interview guides were built on the basic knowledge acquired from the quantitative study and explored respondents’ experiences with IMIS, how they were introduced to it, and how it affected the way they worked. We probed specifically for any perceived facilitators of or barriers to successful adoption, provided the respondents room to elaborate on their expectations for IMIS, and asked them to suggest improvements to the software or ecosystem to improve adoption. We piloted the interview guides with 2 HCWs and 1 experienced manager and modified them according to their insights. After receiving training on qualitative methods, LS and AMM conducted all interviews together in person in July 2019, either in English or in Swahili, with instant translation to English by AMM. Interviews were audio-recorded and transcribed verbatim by LS. With the exception of 4% (1/24) of the participants, interviews were conducted at the workplace of participants. Of the 24 participants, 2 (8%) managers had previous professional relationship with AMM, and the other participants were unknown to the researchers. All the participants were provided an information sheet about the study. The researchers took additional field notes that were used as memos during the analysis stage.

### Data Analysis

Our analysis proceeded in 2 stages and relied on the framework method [38], which was selected because of its structured approach and suitability for multidisciplinary research teams with heterogeneous qualitative experience [39]. First, we coded all transcribed material using a mixture of deductive and inductive coding. We developed a preliminary codebook, integrating elements from the implementation frameworks *Technology, People, Organizations, and Macroenvironmental factors* (TPOM) [40] and *integrated–Promoting Action on Research Implementation in Health Services* (i-PARIHS) [41], into a socioecological model [42], but allowed ourselves to modify codes and include new ones, as we proceeded through the material. Second, we organized codes and related emerging themes according to the framework suggested by Labrique et al [18], which identifies 5 key areas of focus to be considered when pursuing the scale-up of DHIs in LMICs: intrinsic program characteristics, human factors (eg, training, motivation, and support), technical factors (eg, simplicity and interoperability), health care ecosystem (eg, financing and regulation), and extrinsic ecosystem (eg, network, electricity, and hardware) [18]. We trusted that these key areas, developed to be applicable to any DHI, would also be relevant to assess the scalability of DHIs that specifically target health financing.

We purposely adopted different conceptual approaches at different stages of the analysis. First, we turned to the socioecological model [42] for coding because we wanted to ensure the inclusion of all relevant elements at all social levels (ie, individual, interpersonal, organizational, community, and public policy). Hence, we selected a framework that would force us to remain close and true to the data. Later, we relied on the framework by Labrique [18] to organize emerging themes because we felt that this framework was well aligned with the intention of our study, providing a thematically oriented structure that displays how elements acting at each level of the socioecological model play a role to explain successes and failures within each area relevant for scalability.

LS coded the material using NVivo (version 12; QSR International) and with support from MDA. The emergent interpretation and relevance of the findings were discussed among all authors. An audit trail consisting of theme and interview summaries and memos documented the process and all major decisions.

### Ethics Approval

Ethics approval was obtained from the ethics committee of the Heidelberg University in Germany (S-285/2019) and the National Institute for Medical Research in Tanzania (NIMR/HQ/R.8a/Vol. IX/3031). Written informed consent was obtained from all the participants.

## Results

### Study Sample

We conducted 81% (17/21) of the interviews with HCWs (in 16 facilities) and 19% (4/21) of the interviews with district managers, each lasting approximately 60 to 90 minutes. Unfortunately, a district manager passed away shortly before the interview, and hence, the manager of a nearby, similar-performing district in the same region was interviewed instead. In all other cases, the initially sampled interview partners agreed to participate. Table 1 describes the informants’ characteristics.

**Table 1:** Characteristics of participants (N=24)^a^.

Characteristics			HCW^b^ (n=20, 83%), n (%)		Managers (n=4, 17%), n (%)
**Sex**					
	Female	7 (35)		1 (25)	
	Male	13 (65)		3 (75)	
**Age range^c^ (years)**					
	20-29	13 (65)		0 (0)	
	30-39	3 (15)		4 (100)	
	40-49	2 (10)		0 (0)	
	50-59	1 (5)		0 (0)	
**Time in facility or office (years)^d^**					
	<1	4 (20)		1 (25)	
	1-3	8 (40)		3 (75)	
	>3	6 (30)		0 (0)	
**Profession**					
	Clinical officer	8 (40)		N/A^e^	
	Nurse or midwife	11 (55)		N/A	
	Medical attendant	1 (5)		N/A	
**Facility in charge**					
	Yes	13 (65)		N/A	
	No	7 (35)		N/A	

^a^In 1 facility, 2 staff members were interviewed separately because the first interviewee was not primarily responsible for the Insurance Management Information System at the facility. In 3 facilities, a second staff member working with the Insurance Management Information System joined the ongoing interviews, resulting in 24 informants overall.

^b^HCW: health care worker.

^c^The age of 5% (1/20) of the HCWs is unknown.

^d^The time in facility for 10% (2/20) of the HCWs is unknown.

^e^N/A: not applicable.

### Main Themes

Our findings were clustered around the 5 key areas identified by Labrique et al [18]. However, our themes reflect key findings related to each area, in an attempt to stay true to the emic perspective reported by our respondents.

#### Theme 1—Intrinsic Software Characteristics and Technical Factors: IMIS Is Appreciated and Acknowledged as a Valuable Tool by HCWs and Management Alike

The general perception about IMIS was overwhelmingly positive. Even HCWs in facilities struggling with its implementation preferred IMIS to going back to a paper-based claim system. They considered IMIS more as a potential solution to many issues concerning the scheme management at their level and less as a problem:

I think IMIS is perfect. Nothing should be removed or edited.

nurse, 56 years; low adoption level

Most HCWs (18/20, 90%) considered IMIS to be easy to use, at least if one had some previous experience with technology or were motivated to engage with it. The main positive features highlighted were the ability to enter claims offline (claims still had to be uploaded at some point, which requires an internet connection) and the facilitation of fast and smooth business procedures. Digital claiming via IMIS reduced claim processing times, saved travel costs, and eliminated the problem of paper claims getting lost. HCWs appreciated that IMIS would prompt the correction of incorrect client IDs at the time of entry:

The main job of the system is to simplify, and to reduce time of waiting. So when we turn back to the paper, it forces you to take your paper, transport to the council, go back and to wait for results. And then the result can be “come back and take paper number 5. It is not good. Go and feed again, then return back to the council.” So the system [IMIS] is simple.

CO, 24 years; low adoption level

HCWs also acknowledged a more general sense of necessity to transition to digital technologies. For managers, enjoyment and features related to data availability played a major role in their appreciation of IMIS. The ability to produce reports on enrollment or claims with a few clicks made their work visibly easy and more efficient. It further allowed them to specifically approach facilities with a change in claiming patterns, either to congratulate them on the improvement or to offer help when the number of claims was declining:

IMIS makes me feel my work is worth. We have a number of different coordinators in my district that don’t have these systems. I can see how they suffer. IMIS makes me appreciate the office that I sit in. An office without electronic devices to make sure that your duties are simplified, is just not an office.

CHF manager, 28 years; low adoption level; pilot

The only area of IMIS where many saw scope for improvement was feedback on claims. After submitting a claim, the HCW can instantly review whether the claim is accepted or rejected. Although respondents appreciated this feedback, there was a lot of confusion around it. Some (4/20, 20%) were not aware of the feedback at all, and most others (14/20, 70%) did not know that it only represented a first step in the validation process and that initially accepted claims could still be rejected at a later stage. Furthermore, IMIS did not provide any explanation regarding why a claim was rejected. Although claim summary reports could be generated by the CHF management, their sharing with health facilities clearly seemed to be lacking:

But what about the rejected claims? I submitted 100 claims, you reject 30 claims. You just reject without telling me, but I am working for it! I gave medication to them. So the system is like: “whatever, we don’t care.”...Every claim that is rejected, the reason should be highlighted. That I would change.

CO, 28 years; high adoption level

#### Theme 2—Human Factors: The Workforce Does Not Feel Sufficiently Prepared to Integrate IMIS in Their Daily Activities

##### Knowledge and Training

Most respondents (19/24, 79%) did not consider digital literacy as a barrier to adoption because they were used to handling smartphones; however, a few of them complained that older staff members were not willing or able to learn and share the workload associated with IMIS.

HCWs considered insufficient training as a major barrier, with the call for more comprehensive and frequent training sessions emerging across most HCW interviews:

In my opinion, the system was introduced without making any research. Why do I think that? When you establish a system like that, you have to take more time to train the people who are going to deal with the system. A one-day training is not enough, and you can see that not every staff is using the smartphone.

CO, 27 years; high adoption level; pilot

Most HCWs (13/20, 65%) had attended a 1-day or 2-day training session, with only a few having received a 5-day training and 15% (3/20) of them not having received any training at all. In most facilities, only 1 staff member was trained in IMIS. When this person was absent or shifted to a different facility, usually, the claims could not be entered. HCWs considered on-job training as a valuable addition to organized training sessions, but not as an alternative.

##### Support

All HCWs could name the district CHF manager as their reference person for any IMIS-related issues. Although most HCWs (15/20, 75%) considered the availability and responsiveness of managers to be good, the quality of support was only rated as sufficient by HCWs in 3 out of the 4 districts. In the district with the lowest adoption, HCWs complained about their manager’s lack of knowledge about IMIS and their inability to solve their issues:

Even him [the CHF manager], he needs to be getting more experience about it. Because as I said, when you ask him the question, he says: let me ask my superiors. So it’s delaying to ask his superiors and then until the feedback comes back to me it takes a long time and you find you have other responsibilities. This becomes a challenge.

nurse, 40 years; high adoption level

HCWs and managers considered supportive supervision by CHF managers as a crucial element for the success of IMIS. In districts with high adoption, HCWs mostly rated supportive supervision as sufficient, whereas in those with low adoption, visits were either considered as not helpful or were nearly nonexistent:

It is high time for the [CHF manager] to at least have a regular time to visit each health facility to make sure that the facility submitted the claims.

CO, 45 years; low adoption level

All districts had a *WhatsApp* group with HCWs in charge of the facility and CHF district staff, which HCWs considered as an important addition to one-on-one support, facilitating on-the-spot assistance not only by the coordinator but also by IT staff or colleagues from other facilities.

##### Workload

High workload was the most commonly mentioned barrier to the adoption of IMIS. Its introduction added to an already high workload, especially because HCWs still had to fill paper claim sheets for their archives and inspections, resulting in a dual reporting system added to the already existing patient registry and other reporting needs (eg, aggregated versions of similar data to the national health management information system). When queried about why she thinks that claims are not always entered, an HCW summarized as follows:

I think the reason is the workload. The person who is working now, claiming on paper, is the one who is going to enter it into the system later. Is there a person who is capable of working 24 hours?

CO, 26 years; low adoption level

HCWs and managers considered staffing levels to be very low. Most HCWs entered claims after working hours because they could not find the time during the day:

We take for this task extra time. Extra time, you know, is the time to be with the family, but sometimes you will be busy with these claim forms...But it is very difficult to claim during the management because you can’t let wait the client population. So you are supposed to do this after work.

nurse, 30 years; high adoption level

For instance, in June, my fellow wasn’t around. So I have to attend patients, have to attend the clinic, have to attend the labor room, I have to send those claims. So I didn’t send those claims.

nurse, 24 years; high adoption level

Several HCWs (3/20, 15%) suggested the addition of a staff member solely responsible for all IMIS-related or IT-related work as a possible solution for this issue.

#### Theme 3—Extrinsic Ecosystem: Infrastructure Deficiencies Hamper Proper Adoption of IMIS

##### Internet

Respondents considered unreliable internet connection at rural facilities as a key barrier to the adoption of IMIS. None of the facilities had a fiber connection, and all of them relied on mobile internet connectivity. Although some facilities had a consistent mobile network connection, in most facilities, it was unreliable and varied depending on the time of day. This especially became a challenge when connectivity was needed on demand to verify a client’s enrollment status when they walked in. As claim submission could be performed offline and claims could subsequently be uploaded at a different time or place with better internet connectivity, internet issues were not as pressing here. However, HCWs still had to invest time and energy to find sufficient network to upload claims. Managers confirmed that network was a problem, especially for rural facilities:

Sometimes,...we take it to a place in the hills. So we climb the hills so we can access at least the H+.

nurse, 28 years; low adoption level

I always submit them during the night. In our area at night the internet is stronger, almost to E+.

CO, 27 years; high adoption level; pilot

Overall, improving the internet connection was one of the most common suggestions to improve adoption of IMIS.

##### Hardware and Electricity

Respondents did not consider hardware as a major barrier to IMIS adoption. However, the hardware situation differed across districts. In the district with the lowest adoption, HCWs in 3 out of 4 facilities had to use their own phones for IMIS because of a lack of other devices. In another district, every facility had an IMIS-specific phone and a laptop, and all HCWs in that district (4/4, 100%) considered claiming with the laptop to be easier and faster than claiming via phone. However, as the laptop required electricity and internet connection for claim entry, not everyone could use it for claiming. None of the respondents (0/24, 0%) considered the lack of electricity as a barrier to adoption.

#### Theme 4—Health Care Ecosystem: The Insufficient Financial Sustainability of the Insurance Scheme and Lack of Stewardship are Barriers to Adoption

##### Finance

Claim reimbursements were mostly very low, often delayed, and sometimes completely missing. Most HCWs and some managers considered this as a severe barrier to adoption. Several HCWs (7/20, 35%) commented that claiming was not worth the effort, given the small reimbursements. Although many respondents (15/24, 63%) acknowledged that the recent restructuring of CHF (shifting responsibilities to the region) improved the reimbursement situation, it remained as a key barrier:

You took almost 2-3 hours, you are claiming for claims, and you receive 5,000, or 2,000, or 10,000. Somehow, it discourages. So somebody can say, “ah, I leave it.”

nurse, 36 years; high adoption level; in 2019, TZS 1000 was equal to approximately US $0.44] [43

Because if I claim 1,000, then I am paid 500. Why bother? Rubbish.

CO, 45 years; low adoption level

You can’t be doing something that is not paying.

nurse, 24 years; low adoption level

When asked if the facility would have more money available if the CHF did not exist, HCWs provided divergent responses. Although some said they would have more money, others argued that it may be even less, as some clients may not come to the facility without the CHF, and most were exempted from payments.

Managers offered different explanations for the low reimbursement levels. Although some argued that they were appropriate, given the low enrollment in their district, which was reported at 2% to 16%, a manager from a district with low adoption in 2017 reported delays in the payment of the matching grant by the government and that CHF were misused by the district:

...The collected money was deposited to the account of the district. And when the money was being deposited there, it was used by the district. So, if [the CHF] would request the fund for the dispensary, the money was not being paid.

CHF manager, 34 years; low adoption level

The wide health financing context also influenced the adoption of IMIS. In the region with low adoption, there was a results-based financing (RBF) scheme, funded by United States Agency for International Development and implemented by the World Bank [44], paying premiums to facilities based on achieving predefined quality targets. This scheme was reported to pay a lot more money than the CHF, and HCWs considered it as a more relevant source of funding for them, even though some indicated that, being a donor fund, it was an unsustainable financing source. Some suggested that IMIS claiming may have been disadvantaged by the presence of RBF:

The facility may ignore [claiming] because of the presence of the RBF and the basket fund. Because the RBF is present, they think that they don’t have to do it. Intentionally they don’t do it.

CHF manager, 39 years; high adoption level

RBF and the basket fund are a project, donor funds, they don’t sustain for long. But CHF is our own money, our own system, it will stay for long.

CHF manager, 39 years; high adoption level

Another financial aspect that respondents considered as a barrier to adoption was the fact that most HCWs paid for the internet necessary for claim upload with their own personal money. In combination with the low reimbursements, this was seen as a potential reason for not submitting claims. Although managers argued that facilities could include the costs in their budget, only 4 facilities were aware of this possibility:

It’s challenging. It’s difficult for me to let’s say buy a bundle for 2.000 and then my children are starving at home.

CO, 45 years; low adoption level

##### Stewardship

CHF managers considered stewardship by the district health authorities to be crucial for the successful implementation of CHF and IMIS. Although CHF managers are stewards on the purchaser side, all managers (4/4, 100%) highlighted that on the service provision side, the district medical officer (DMO) could directly influence facility-claiming behavior or that their initiatives affected the financial sustainability of the scheme. It is notable that managers of districts with high adoption levels in 2017 reported effective DMO stewardship, whereas those with low adoption levels mentioned problems in cooperation, with a manager experiencing a recent change:

The CHMT [Council Health Management Team] was a problem toward pushing CHF system, especially pushing the IMIS programs operation. They were like “it is another activity which has been introduced to the facilities, which is out of our scope.” But after understanding now, they are coping it...Our district was getting very few money for claims. But since his [the DMO’s] initiatives, we started getting at least a certain amount. We started with 100,000. Last month we received 1.3 million. Now we are progressing, and his initiatives are working.

manager, 32 years; low adoption level

We are not working with him [the DMO] effectively. That’s why others don’t claim.

manager, 34 years; low adoption level

## Discussion

Our study explored the *why?* behind the low adoption of IMIS by HCWs in rural regions of Tanzania. It offers insights into the adoption-related factors that should be considered for a successful scale-up of digital solutions for health financing, linking existing knowledge on DHIs in LMICs to the field of health financing.

### Findings in Context

HCWs and managers appreciated the intrinsic software characteristics of IMIS (theme 1) and considered the software to be easy to use and useful to facilitate more efficient claiming. This is a promising finding because a swift claiming mechanism is key to the implementation of strategic purchasing [36,45]. Our findings suggest that IMIS could—in principle—facilitate this.

The ability to enter claims offline, store them on the phone, and upload them at a different time or place facilitated claiming or made it possible in the first place at some facilities. However, as observed in other contexts previously [46], offline functionality could not solve all issues arising from limited internet availability because CHF membership verification still required on-the-spot internet availability. Establishing a membership verification mechanism that does not rely on internet, for example, via SMS text messaging or unstructured supplementary service data (USSD), a technology that has been successfully used in a number of health-related programs in resource-limited settings [47,48], could partially solve this problem [18].

Managers made extensive use of IMIS’s data inspection functions, unveiling its potential in facilitating real-time data management. HCWs, as end users of the software, were not able to profit from these owing to limited claim feedback and lack of training. Improving HCWs’ abilities to view the outcome of their work (decision on claim submissions), understand mistakes, and use the data they enter into IMIS may give them a more immediate sense of usefulness or joy and help to improve adoption. Fostering a data use culture among end users, as routinely recommended [2,19], has been shown to benefit DHI adoption in a study on an electronic immunization registry in Tanzania, Zambia, and elsewhere [49,50].

Human resource constraints are a key weakness of health systems in LMICs [49,51-54]; therefore, it is unsurprising that human factors (theme 2) such as workload, training, and support were among the most commonly discussed barriers to successful widespread adoption of IMIS. In Tanzanian health facilities, understaffing is a widespread problem [31,55] that poses challenges to many health system interventions, including DHIs [49]. Half of the facilities we visited had only 2 staff members, and they were well below the government recommendations [32]. At the core, understaffing is a broad health system problem and can only be solved—flanked by other measures—by increased financial investments. When judging IMIS’s influence on workload, HCWs seemed torn between acknowledging the increased speed of business procedures on the one hand and added workload for claim entry on the other hand. This reflects the heterogeneity in the literature, where DHIs have been reported to save time in some scenarios and increase workload in other scenarios [17,49,56]. Other health financing interventions such as RBF have also struggled with added workload through additional reporting [57].

In our study, inefficient reporting played an important role in the perceived increase of workload. HCWs had to enter client information in at least 3 separate ways (patient registry, offline claim form, and IMIS). Regarding CHF, transitioning to a digital-only reporting system could reduce this administrative burden and improve IMIS adoption, as this has been observed for other scaled-up DHI implementations previously [23,58]. However, in a general DHI implementation context and especially for solutions attempting to introduce strategic purchasing, where data reporting from facilities is key, a single data entry point, irrespective of who the data go to, in what form, or for which purpose, would be ideal and could improve DHI adoption [59].

Insufficient training and support have also been described as important barriers to DHI scale-up previously. The combination of a low number of HCWs trained in IMIS per facility (usually only 1) and high staff turnover was particularly notable in our study. Both of these issues have been identified as barriers to scale before [23,60], and when combined, they make it nearly impossible to assure the continuous presence of trained staff. A study on an electronic immunization registry in Tanzania showed a direct positive correlation between the number of HCWs trained at a facility and adoption of the DHI [58]; therefore, it is likely that training more staff in IMIS will improve adoption also. As observed previously [49], the use of web-based chat groups (*WhatsApp*) for on-the-spot support by peers or district staff was a facilitator of IMIS adoption. This is particularly interesting for DHIs revolving around mobile phones because the required hardware and the knowledge and skills to use the communication software are already available.

The extrinsic ecosystem (theme 3) played a major role as a barrier to scale in the form of limited internet coverage. This is not surprising, because infrastructure deficiencies have proven to be a key barrier to health system strengthening initiatives previously. As long as basic infrastructure requirements are not met, the success of any intervention—digital or nondigital and related or unrelated to health financing—is severely impeded [2,61]. Lack of internet access being the main infrastructural barrier in our study is plausible for a software relying heavily on web-based data transfer. It is also discussed in most of the literature on DHI implementations in LMICs [11,18,23,50,53,56,62-64]. Our findings further highlight the need for reliable internet infrastructure as a key facilitator of successful DHI scale-up.

The health care ecosystem (theme 4) axis was mainly represented by the insufficient financial sustainability of the insurance scheme within which IMIS was implemented. Respondents’ insights about this aligned well with existing evidence on the financial restrictions of CHF in Tanzania [65,66]. Although IMIS has technologically enabled the management of a new revenue source for the health sector, the financial restrictions of CHF meant that the benefits of facilitating a quick and easy claiming and reimbursement process were often not visible to HCWs, thus reducing the motivation to claim and resulting in low adoption rates. This problem is not unique to CHF, as the negative effects of low or untimely reimbursements have been observed as a key barrier to the success of health financing interventions such as RBF previously [67,68]. Changes in the payment system and formulas also require time for internalization, which could be a reason for the incomplete or limited data entry expressed by participants, as interviews were conducted at a point immediately after a formula change was undertaken in iCHF.

The presence of a donor-funded RBF scheme may have impeded IMIS adoption and thereby CHF performance in a region. This may be an explanation for the low IMIS adoption rates observed in this region in our previous study [27]. Although this has nothing to do with IMIS or DHIs, it shows how the parallel introduction of different health financing approaches or projects in the same facility or administrative area can negatively affect their success.

Our findings on stewardship are particularly interesting because they aligned well with district adoption levels in the previous study. Stewardship is a crucial element of success for the scale-up of DHIs [11,49,69] and for health financing reform [70]. Our findings highlight that support from district authorities can have a major influence on the adoption of DHIs by HCWs.

### Methodological Considerations

Building on the findings from our previous study allowed us to implement purposive maximum variation sampling and eased the interpretation of findings, as we could anchor emerging knowledge to a previously acquired understanding of the context and its dynamics. To minimize the bias that could have arisen from the time interval between the quantitative and qualitative studies, before conducting interviews, we cross-checked that the selected facilities would still display the same adoption levels detected by the quantitative assessment in 2017.

Furthermore, we need to acknowledge that our sample only reflects the experience of rural primary-level facilities, even though IMIS is used at all levels of the Tanzanian health system. However, our choice was deliberate because we considered adoption at this level of care to be the most crucial for scale. Although high-level facilities usually struggle less with the adoption of a DHI [53,58], most are primary-level facilities, and most patient contacts occur at the primary level. Therefore, the success of a DHI scale-up relies heavily on adoption in these facilities.

A consideration to make is that our interviews were conducted a few months after a major change in the CHF design. This timing required us to probe the interviewees carefully for any difference after the change was implemented. Many interviewees (15/24, 63%) acknowledged that some aspects of the themes discussed previously (eg, reimbursements) improved after the reorganization. It is reasonable to believe that with more time, some aspects further improved, as any adjustment of a project implementation needs time to develop to its full potential. However, as the general notion of interviewees was that the same problems persisted after the reorganization, only some with different severity, we are confident that our results are valid even after more time has passed.

Finally, we wish to consider that as our study was based only on 2 regions in 1 country, the reader needs to gauge transferability to other settings within and beyond Tanzania based on their own assessment of the extent to which the contextual factors we describe capture system elements that are relevant also beyond our study setting. In general, we trust that, given the wide applicability of IMIS across LMICs, our findings can inform implementation policies beyond our study regions.

### Conclusions

Our study shows that IMIS may have the potential for scale, as it is valued by the end users. However, careful consideration should be given to the environment in which it is implemented. A sustainable health financing environment, sufficient infrastructure, and human capacity are prerequisites for successful scale-up and were perceived as severely flawed by HCWs and managers in the context of IMIS in Tanzania. If these are not accounted for, digital interventions for health financing may not be able to reach scale or unfold their potential to improve core health financing functions such as enabling strategic purchasing and will only have limited potential to contribute to UHC. Studies from other settings are needed to identify the best practices for the establishment of these prerequisites.

## Appendix

Multimedia Appendix 1 Guide for health care worker interviews.

Multimedia Appendix 2 Guide for manager interviews.

## Abbreviations

CHF: Community Health Fund
CO: clinical officer
DHI: digital health intervention
DMO: district medical officer
HCW: health care worker
HPSS: Health Promotion and System Strengthening
iCHF: improved Community Health Fund
IMIS: Insurance Management Information System
i-PARIHS: integrated–Promoting Action on Research Implementation in Health Services
LMIC: low- and middle-income country
RBF: results-based financing
TPOM: Technology, People, Organizations, and Macroenvironmental factors
UHC: universal health coverage
USSD: unstructured supplementary service data

## Data Availability

The data sets generated and analyzed during this study are available from the corresponding author upon reasonable request.
