# Supplementary material for: Factors Affecting the Successful Implementation of a Digital Intervention for Health Financing in a Low-Resource Setting at Scale: Semistructured Interview Study With Health Care Workers and Management Staff
Source: J Med Internet Res. 2023 Jan 6;25:e38818. doi: 10.2196/38818 (PMC9862332; doi:10.2196/38818)
Supplement: Multimedia Appendix 1 [file jmir_v25i1e38818_app1.pdf]

This table is to be filled in in the field, and to appear at the top of each transcript	
**Interview Details**	
Date (Year- Month- Day-)	
Region	
District	
Facility	
**Participant Details**	
Gender	
Position/cadre	
Age	

Interview Guide for semi-structured interviews with health care workers on the implementation of IMIS within the "improved CHF" in Tanzania

1. Think about your job here at [FACILITY] in [DISTRICT NAME], in terms of the work that you do.
    a. What is your job title and how long have you been in your current position?
    b. What are your daily tasks?
    c. How many clients do you see in a month/week/day? Estimate, no need to look up.

2. Before we talk about IMIS, I would like to discuss your knowledge, experiences and opinion about the Community Health Fund (CHF).
    a. Have you heard about the CHF and can you briefly try to explain what it is in your own words?
    b. Is this facility part of CHF? Since when? How many of your clients are CHF enrolees? On average, how many CHF enrolees do you see in a month? How many patients overall?
    c. Please explain to me as precisely as possible what happens when a CHF client walks into this facility and seeks treatment. Please focus on the administrative part of patient registration, documents filed, payments made etc. We don't have to discuss the medical treatment.
    d. Does this facility offer any services that are not covered by the CHF?

3. I wonder at which point you receive money for the treatment. Could you please elaborate? (prompt the following if necessary)
    a. Do you receive money from the CHF for treatments of CHF insured patients?
    b. Does this money come in on a regular basis?
    c. How do you feel about the amount paid to you by the CHF? Is it appropriate?
    d. Do you receive any payments from CHF insured patients?
    e. Is there any other way for your facility to receive money for CHF patients other than through IMIS entries or the options discussed above?
    f. If the CHF would not exist and everyone had to pay the fee, would you receive more money overall? (prompt: exempted)

4. From your perspective, is the CHF a good system? Does it work in your eyes?
   Please explain your opinion and use examples if they help you explain.

5. For the rest of our conversation, I would like to focus on IMIS as a tool in the CHF.
    a. Do you use IMIS as part of your job? If so, how? With what frequency? For what functions? (Obtain detail on work done with IMIS)
        i. Prompt CHF membership enquiry
    b. How long have you worked with IMIS now? Always for the same tasks and in the same frequency?
    c. Have you worked with IMIS before at another facility or in another job? Where? For how long? What were your tasks?

6. How was IMIS introduced to you?
    a. Who explained it to you?
    b. How did they explain it to you?
    c. How do you understand its purpose? (has that understanding evolved over time?)
    d. Did you receive any specific training? Like seminars, online courses?
    e. Do you have an assigned reference person for IMIS related questions and where is that person located?
    f. Does that reference person visit the facility? How often?
    g. Has integrating IMIS in your daily work been easy or not? Please explain, referring both to yourself and to the facility as a whole.

7. Now please tell me how you, as a [JOB TITLE] at [FACILITY NAME], work with IMIS.
    a. How much time does your work related to IMIS consume?
    b. How does IMIS affect your work life in terms of daily routines?
    c. Do you feel you have enough time for the work associated with IMIS?

8. I am interested in the procedures at this facility with regard to IMIS.
    a. Are multiple people in charge of entering data in IMIS or only one?
    b. Who else enters data in IMIS? (Job titles)
    c. Who is responsible for filling out paper-based claim sheets at your facility? Are paper-based claim sheets always clearly understandable to you? Can you easily check with the person responsible to dissolve problems?
    d. If you are away, e.g. sick for a longer period, is there someone else who can take up the job of entering data into IMIS?
    e. How often are data entered and how? (mobile/online/offline/paper sent to district officer)
    f. Can you describe to me precisely what information is entered into IMIS and how? With what purpose? (prompt showing an example here if possible, see last question)
    g. Do you receive any kind of feedback on the data entered? By whom? With what frequency? On what items? With what purpose? (prompt rejected claims)
       Are you happy with the feedback you get? What else would you like?
    h. Is using IMIS an easy or a complex task? Why? Can you explain your position with some examples?

9. Now I would like to talk about your experiences and expectations in relation to IMIS and how these expectations have evolved over time.
    a. First, I want to talk about the purpose of IMIS as understood by you.
        i. How did you first understand the purpose of IMIS when it was introduced to you? (or when you started working here)
        ii. How has this understanding evolved?
        iii. How do you understand its purpose today?
    b. Please describe some positive effects brought by IMIS.
    c. Please describe some negative effects brought by IMIS.
    d. How do you feel to these effects as they relate to your work?
    e. In your opinion, how important is it that CHF patients who visit the facility are entered into IMIS? Please explain why you think that way.

10. Continuing with your personal opinions, I would like to focus on challenges you see with regards to IMIS and the way they are addressed. Please keep in mind that there are no right or wrong answers, and you are not obliged to answer if you feel some of the questions are not relevant to you.
    a. From your point of view, what were challenges that you and other staff at the facility faced in taking up use of IMIS? Are these challenges persisting?
    b. How would you describe how your facility addressed these challenges?
        i. What did you and/or your colleagues in the facility do to address these challenges? Please share an example and be as specific as possible.
        ii. What, if any, are some of the factors, elements and/or people that made it possible to resolve these challenges?
        iii. What, if any, are some of the factors, elements and/or people that made it infeasible to resolve these challenges?
    c. If you or your colleagues in the facility cannot resolve a problem, do you approach a reference person? How would you judge the availability of this reference person?

11. I have found that in many facilities, not all claims are entered. What, in your experience, motivates this?
    a. Not efficient?
    b. Lack of knowledge about the scheme? Lack of skills?
    c. Lack of time? Workload?
    d. Internet connection?
    e. No money received? ...
    f. Can you imagine that any of these are also an issue for this facility?

12. One last question: Imagine that you are asked to change one or two things about IMIS *or* about some facet of your work for it to be more amenable to IMIS. Looking ahead, please

tell me in as much detail as possible what you see as the most important factors to improve IMIS *or* your facility`s workflow with regard to IMIS.

a. Can you imagine any changes to the way IMIS is implemented in this facility that would improve the situation?
b. What would you do different if you had the power to?
c. What would make your work with IMIS easier personally?
d. Would you prefer if IMIS would be removed? (i.e. back to paper-based claims)

13. Is there anything I should have asked you about IMIS or anything else that you could add?

14. Before we end this interview, I would like to ask you one more thing if this is possible. I would like to see how an entry into IMIS is actually done. So if you agree and if it is possible right now, would you demonstrate how a patient is entered into IMIS? (if not done before)

15. Any final thoughts?

Thank you

Afterwards, look into:
Claim book
Patient registry
