# Supplementary material for: Factors Affecting the Successful Implementation of a Digital Intervention for Health Financing in a Low-Resource Setting at Scale: Semistructured Interview Study With Health Care Workers and Management Staff
Source: J Med Internet Res. 2023 Jan 6;25:e38818. doi: 10.2196/38818 (PMC9862332; doi:10.2196/38818)
Supplement: Multimedia Appendix 2 [file jmir_v25i1e38818_app2.pdf]

This table is to be filled in in the field, and to appear at the top of each transcript	
**Interview Details**	
Date (Year- Month- Day-)	
Region	
District	
Facility	
**Participant Details**	
Gender	
Position/cadre	
Age	

Interview Guide for semi-structured interviews with iCHF management staff on the implementation of IMIS within the "improved CHF" in Tanzania

1. Think about your job here as CHF manager in [DISTRICT NAME], in terms of the work that you do.
    a. How long have you been in your current position?
    b. What are your daily tasks?

2. Before we talk about IMIS, I would like to discuss your knowledge, experiences and opinion about the Community Health Fund (CHF).
    a. Can you briefly try to explain what the CHF is in your own words? No need to go into the history.
    b. Do you have an idea of how many people are insured via the CHF in this district? On average, how many CHF enrolees visit a health facility/dispensary in a month?
        i. Significant changes from 2017 to today?

3. From your perspective, is the CHF a good system? Does it work in your eyes?
   Please explain your opinion and use examples if they help you explain.

4. I wonder at which point the facilities receive money for the treatment. Could you please elaborate and focus on dispensaries?
   (prompt the following if necessary)
    a. How often is money paid out?
    b. How much of the collected money is paid out to facilities?
    c. Is there enough money to pay out?
    d. How do you feel about the amount paid to dispensaries? Is it appropriate?
        i. Is there any other way for facilities to receive money for CHF patients other than through IMIS entries or the options discussed above?
           prompt: informal payments? Paper based system?

5. For the rest of our conversation, I would like to focus on IMIS as a tool in the CHF.
    a. Do you use IMIS as part of your job? If so, how? With what frequency? For what functions? (Obtain detail on work done with IMIS)
    b. How long have you worked with IMIS now? Always for the same tasks and in the same frequency?
    c. Have you worked with IMIS at another job before? Where? For how long? What were your tasks there?
    d. What was your last occupation before you started working as a CHF manager in this district?

6. How was IMIS introduced to you?
    a. Who explained it to you?
    b. How did they explain it to you?
    c. Did you receive any specific training? Like seminars, online courses?
    d. Do you have an assigned reference person for IMIS related questions and where is that person located?
    e. Are you yourself a reference person for IMIS related questions for others or do you give special training for people working with IMIS?
        i. Do you visit facilities? How often?
        ii. Can you name some examples of questions you have to answer as a reference person
    f. Has integrating IMIS in your daily work been easy or not? Please explain, referring both to yourself and to your district/region.
    g. Is it an easy tool
        i. How long did it take you to become competent in IMIS?

7. I am interested in the procedures at your district with regard to claim entry in IMIS.
    a. Do facilities in your district generally enter their own claims? From the top of your head, can you give an estimate of how many entries facilities enter on their own and how many paper claims are sent to you to enter into IMIS?
        i. Changes from 2017?
    b. If a paper claim is sent to your office:
        i. Is it necessary to enter every claim into IMIS or are there other ways to record the claim for reimbursement?
        ii. Are multiple people in charge of using IMIS or only one?
        iii. Who else enters data in IMIS?
        iv. If you are away, e.g. sick for a longer period, is there someone else who can take up the job of entering data into IMIS?
        v. How often are data entered and how? (mobile/online/offline)
        vi. Do you receive any kind of feedback on the data entered? By whom? With what frequency? On what items? With what purpose? (depending on position)
        vii. Do you give any kind of feedback on paper claims sent to you? To whom? With what frequency? On what items? With what purpose?
    c. If a claim is entered by the facility:
        i. Is it common that there are mistakes in the entries?
        ii. Do these mistakes lead to rejection of claims?
        iii. Do you give any kind of feedback on claims entered by facilities? To whom? With what frequency? On what items? With what purpose?

        iv.

8. Now please tell me how you, as a district CHF manager at [DISTRICT NAME], work with IMIS.
    a. How much time does your work related to IMIS consume? Is entering claims a significant part of that time?
    b. How does IMIS affect your work life in terms of daily routines?
    c. Do you feel you have enough time for the work associated with IMIS?

9. Now I would like to talk about your experiences and expectations in relation to IMIS and how these expectations have evolved over time.
    a. First, I want to talk about the purpose of IMIS as understood by you.
        i. How did you first understand the purpose of IMIS when it was introduced to you? (or when you started working here)
        ii. How has this understanding evolved?
        iii. How do you understand its purpose today?
    b. Please describe some positive effects brought by IMIS.
    c. Please describe some negative effects brought by IMIS.
    d. How do you feel to these effects as they relate to your work?
    e. In your opinion, how important is it that claims are entered into IMIS? Please explain why you think that way.

10. Continuing with your personal opinions, I would like to focus on challenges you see with regards to IMIS and the way they are addressed. Please keep in mind that there are no right or wrong answers, and you are not obliged to answer if you feel some of the questions are not relevant to you.
    a. From your point of view, what were challenges that you and other staff at your level faced in taking up use of IMIS? Are these challenges persisting?
    b. How would you describe how you and your colleagues addressed these challenges?
        i. What did you and/or your colleagues do to address these challenges? Please share an example and be as specific as possible.
        ii. What, if any, are some of the factors, elements and/or people that made it possible to resolve these challenges?
        i. What, if any, are some of the factors, elements and/or people that made it infeasible to resolve these challenges?
    c. If you or your colleagues cannot resolve a problem, do you approach a reference person? How would you judge the availability of this reference person?
    d. Can you think of challenges that could not be resolved?

11. I have found that in many districts, not all data are entered. What, in your experience, motivates this?
    a. Not efficient?
    b. Lack of knowledge about the scheme?
    c. Lack of time?
    d. No money received? ...

12. One last question: Imagine that you are asked to change one or two things about IMIS *or* about some facet of your work for it to be more amenable to IMIS. Looking ahead, please tell me in as much detail as possible what you see as the most important factors to improve IMIS *or* your team's workflow with regard to IMIS.
    a. Can you imagine any changes to the way IMIS is implemented that would improve the situation?

b. What would you do different if you had the power to?
c. What would make your work with IMIS easier personally?

13. Is there anything I should have asked you about IMIS or anything else that you could add?

14. Before we end this interview, I would like to ask you one more thing if this is possible. I would like to see how an entry into IMIS is actually done. So if you agree and if it is possible right now, would you demonstrate how a patient is entered into IMIS?

15. Any final thoughts?

Thank you
